# Case Report: Rapidly fatal hepatic portal venous gas following transarterial chemoembolization for hepatocellular carcinoma

**DOI:** 10.3389/fonc.2026.1951000

**Published:** 2026-10-19

**Authors:** Renshan Zhao, Bin Xie

**Affiliations:** Department of Intervention, Linyi People’s Hospital, Linyi, Shandong, China

**Keywords:** case report, drug-eluting microsphere, hepatic portal venous gas, hepatocellular carcinoma, iodized oil, transarterial chemoembolization

## Abstract

Hepatic portal venous gas (HPVG) is an uncommon radiological sign whose clinical importance depends on the underlying disorder and the patient’s physiological state. Only two cases of HPVG following transarterial chemoembolization (TACE) have been reported. We describe a rapidly fatal episode in a 71-year-old man with chronic hepatitis B and biopsy-confirmed hepatocellular carcinoma. Selective TACE involved lobaplatin infusion, followed by 10 mL of an iodized oil-raltitrexed emulsion and 1 mL of epirubicin-loaded drug-eluting microspheres. Constipation and abdominal distension developed approximately 21 h after TACE. Severe abdominal pain and vomiting followed at 37 h. Emergency CT at approximately 38 h showed peripheral branching HPVG, bowel dilatation, and intraluminal fluid. At approximately 39 h, the patient developed impaired consciousness, hypotension, and bradycardia. He died despite resuscitative treatment approximately 40 h after TACE. Lactate, a repeat leukocyte count, blood cultures, and other microbiological samples were not obtained after the acute deterioration. Sepsis therefore remained an unconfirmed possible mechanism. The combined embolization regimen represents a procedural exposure, not a proven cause. Progressive abdominal symptoms after TACE should prompt urgent evaluation for bowel injury, infection, biliary complications, and circulatory deterioration.

## Introduction

Gas within the portal venous system is an imaging finding rather than a diagnosis with a uniform prognosis. HPVG may accompany self-limited bowel distension, intestinal ischemia, or severe infection. Clinical outcome is determined mainly by the underlying disorder and the degree of physiological compromise (1–4).

HPVG after TACE is exceptionally uncommon. The literature contains only two reports: one fatal case with a culture-positive liver abscess and one conservatively managed case with pre-existing pneumatosis and bowel dilatation (5, 6). Neither case involved combined embolization with iodized oil and drug-eluting microspheres. We report a rapidly fatal course after this combined procedure. The treatment combination is reported as clinical context because the available evidence does not establish it as a cause or risk factor for HPVG.

## Case description

### Patient information and clinical findings

A 71-year-old man presented with mild epigastric discomfort one week after receiving a diagnosis of hepatocellular carcinoma. He had chronic hepatitis B for more than 10 years without antiviral treatment and reported a 30-year history of alcohol consumption. He denied hypertension, diabetes mellitus, coronary artery disease, chronic bronchitis, cholelithiasis or cholecystitis, previous surgery or trauma, blood transfusion, and drug or food allergy. He had never smoked. His family history was negative for similar or known hereditary disease. Serum alpha-fetoprotein was within the normal range, and liver function was Child-Pugh class A.

### Diagnostic assessment

At a local hospital, contrast-enhanced abdominal CT had indicated a malignant hepatic tumor. Percutaneous liver biopsy subsequently confirmed hepatocellular carcinoma. Histological morphology and immunohistochemistry supported the diagnosis. Tumor cells were positive for Glypican-3 and glutamine synthetase, focally positive for HepPar-1, and negative for CK7, CK19, CD10, and HSP70. The Ki-67 labeling index was approximately 10%, and CD34 highlighted vascular structures. Reticulin staining was positive. At our hospital, contrast-enhanced MRI showed a 57 mm x 63 mm hypervascular mass in the right hepatic lobe. An additional hypervascular nodule was present in the right posterior segment (**Figure 1**). In this at-risk patient, the imaging features also supported hepatocellular carcinoma (7, 8).

**Figure 1:**
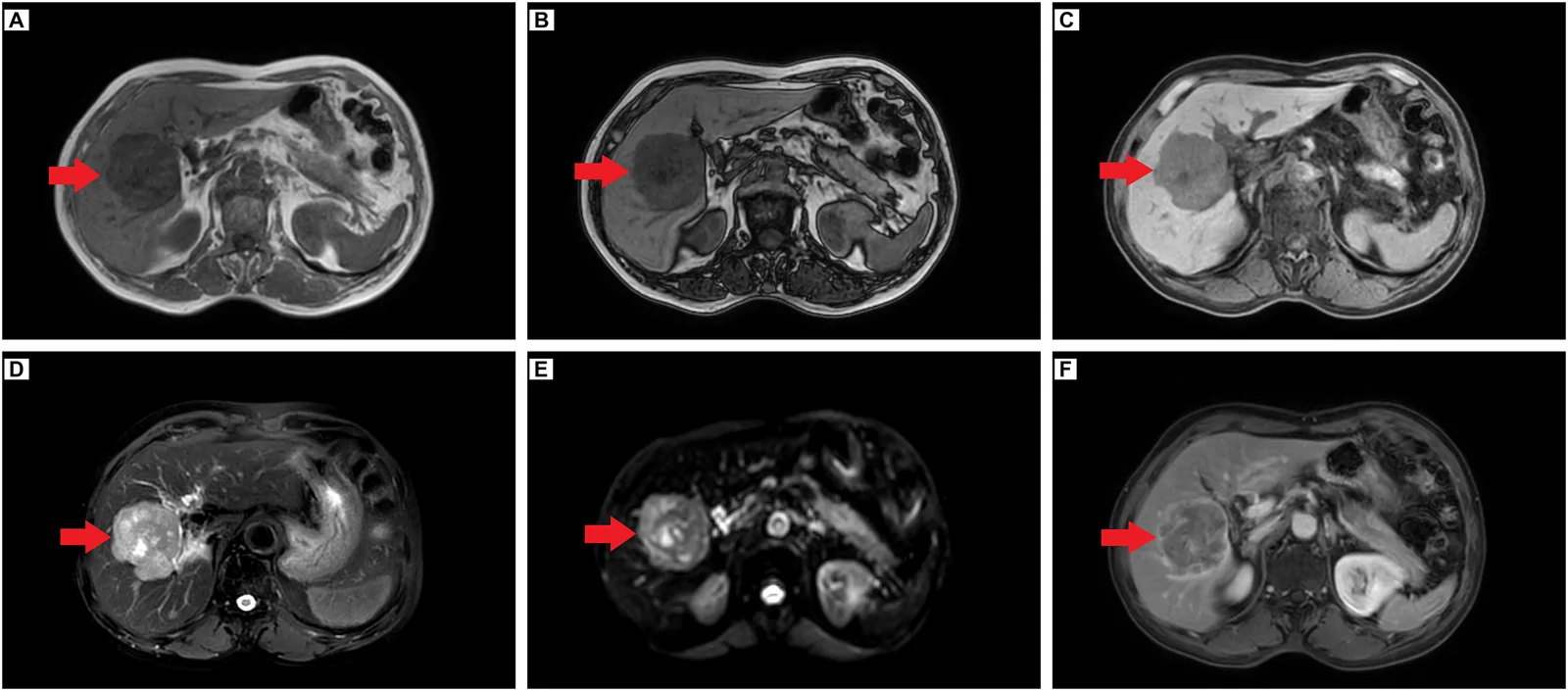
Preprocedural magnetic resonance imaging. Axial MRI images show the right hepatic lobe tumor (red arrows) on unenhanced and contrast-enhanced sequences. **(A)** T1-weighted image with preserved fat signal. **(B)** Opposed-phase T1-weighted image. **(C)** Fat-suppressed T1-weighted image. **(D)** T2-weighted image. **(E)** Diffusion-weighted image. **(F)** Contrast-enhanced delayed-phase image.

### Timeline

**Figure 2** presents the documented clinical course using time elapsed from completion of TACE. It separates clinical findings and imaging from treatment, outcome, and unavailable investigations.

**Figure 2:**
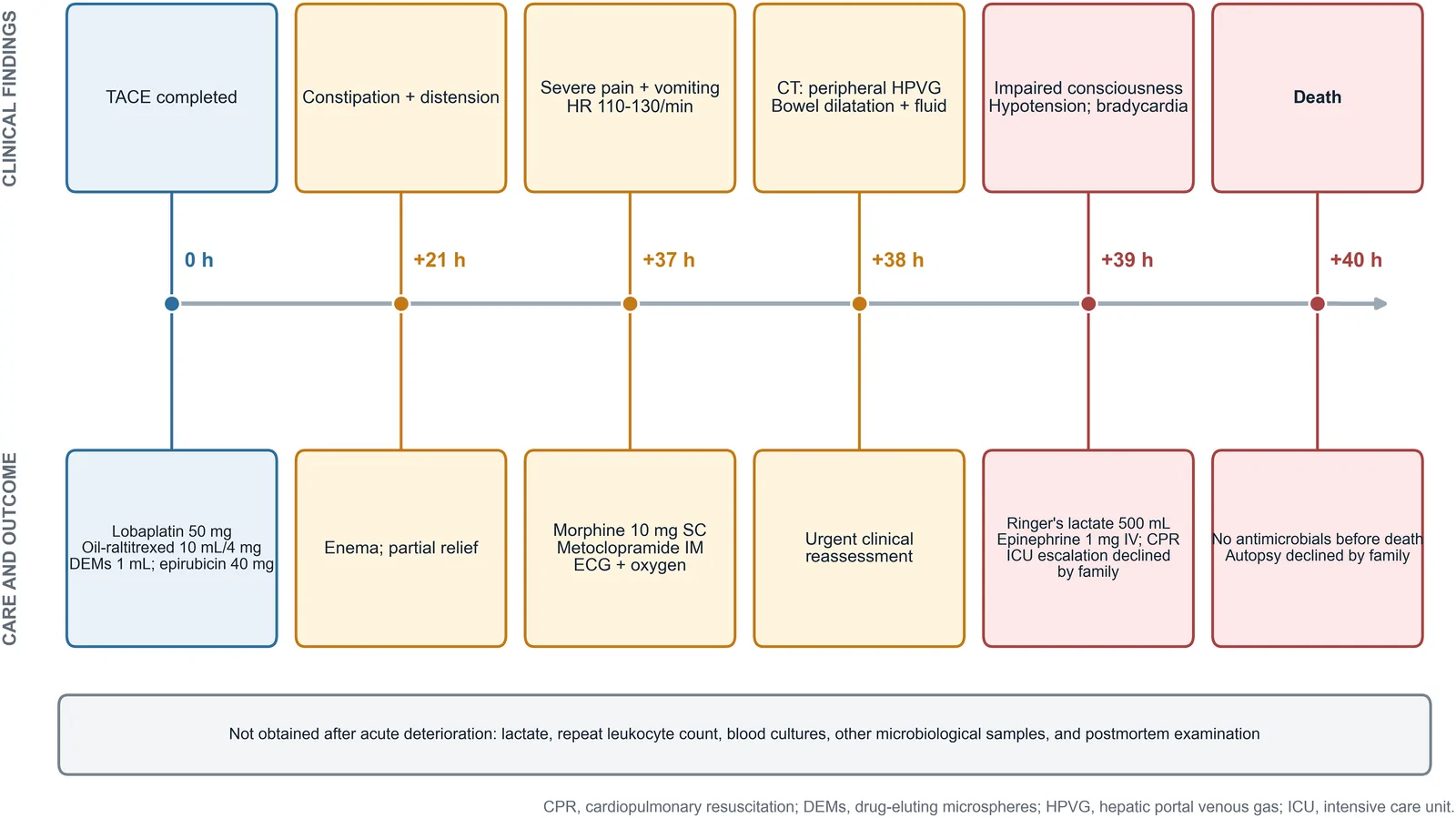
Clinical timeline after transarterial chemoembolization. Relative times are measured from completion of TACE. The upper row shows symptoms, vital signs, and imaging findings; the lower row shows treatment and outcome. Constipation and abdominal distension preceded the enema at approximately 21 h. Severe abdominal pain and vomiting occurred at approximately 37 h, followed by CT confirmation of HPVG at 38 h and circulatory deterioration at 39 h. Death occurred at approximately 40 h. Lactate, a repeat leukocyte count, blood cultures, other microbiological samples, and postmortem examination were unavailable. CPR, cardiopulmonary resuscitation; DEMs, drug-eluting microspheres; HPVG, hepatic portal venous gas; ICU, intensive care unit; TACE, transarterial chemoembolization.

### Therapeutic intervention

Angiography during TACE showed tumor-supplying branches arising from the tortuous right hepatic artery (**Figures 3A, B**). Lobaplatin (50 mg) was infused selectively. Chemoembolization was then performed with an iodized oil-raltitrexed emulsion containing 10 mL of iodized oil and 4 mg of raltitrexed. This was followed by 1 mL of 100-300 μm drug-eluting microspheres loaded with epirubicin (40 mg). Completion angiography showed reduced tumor blush (**Figures 3C, D**).

**Figure 3:**
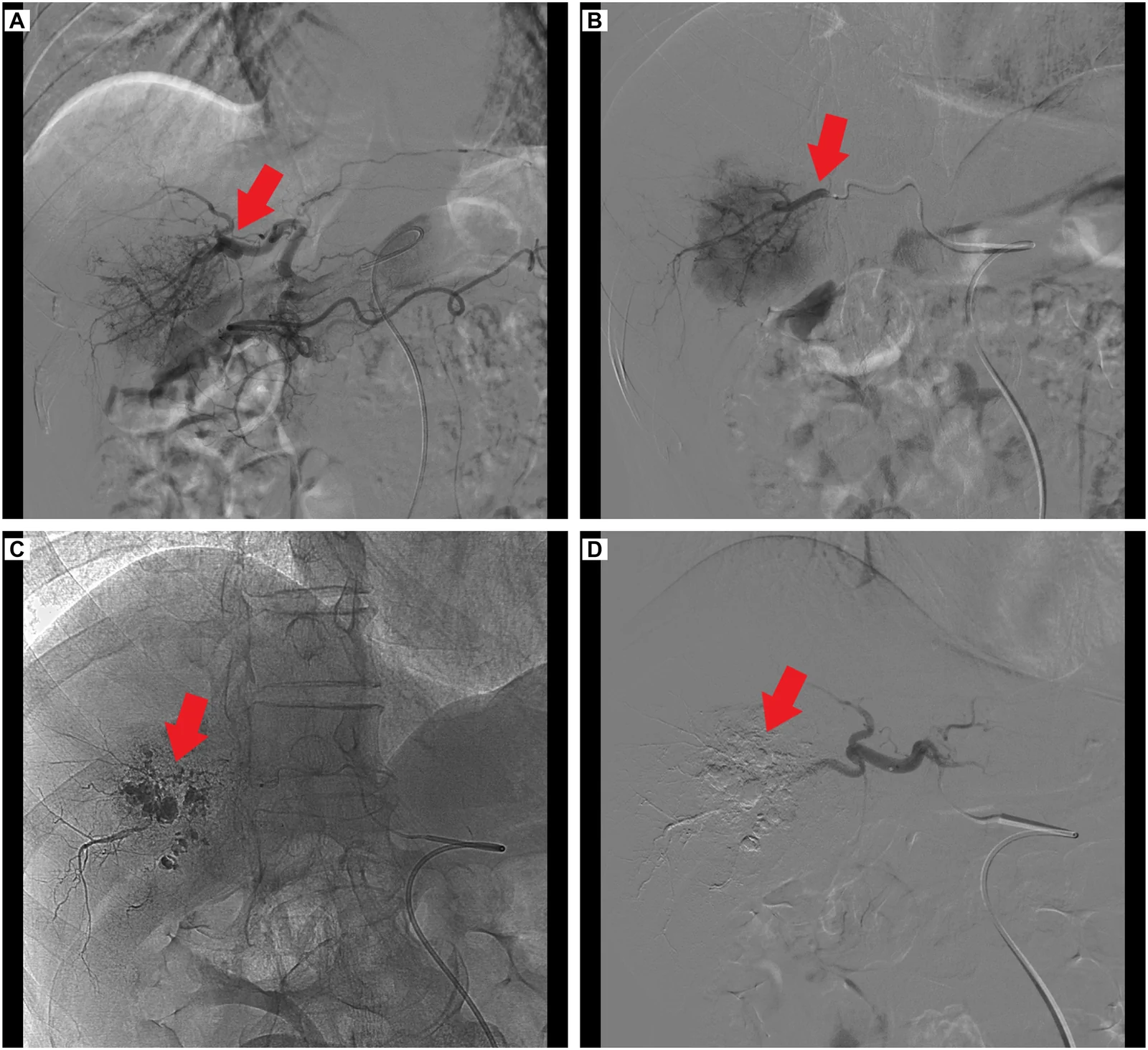
Digital subtraction angiography during TACE. **(A, B)** Pre-embolization angiography shows tumor-supplying branches arising from the tortuous right hepatic artery (red arrows). **(C, D)** Postembolization images show dense embolic-material deposition and reduced tumor vascularity (red arrows).

### Follow-up and outcomes

Approximately 21 h after TACE, the patient developed constipation and abdominal distension, which partially improved after an enema. At approximately 37 h, severe abdominal pain and vomiting developed, with a heart rate of 110–130 beats min-1. Morphine 10 mg was administered subcutaneously, and metoclopramide was administered intramuscularly. Electrocardiographic monitoring and supplemental oxygen were also provided. The symptoms improved transiently. Emergency CT at approximately 38 h showed peripheral branching gas extending to within 2 cm of the hepatic capsule, consistent with HPVG. Bowel dilatation and intraluminal fluid were also present. Additional intratumoral foci may have represented gas mixed with embolic material (**Figure 4**). At approximately 39 h, the patient developed impaired consciousness, hypotension, and bradycardia. Resuscitation included rapid infusion of 500 mL of Ringer’s lactate, intravenous epinephrine 1 mg, and cardiopulmonary resuscitation. These measures did not reverse the deterioration. Lactate, a repeat leukocyte count, blood cultures, and other microbiological samples were not obtained after the acute deterioration. No antimicrobial therapy was administered before death. The family declined transfer to the intensive care unit and further intensive treatment, and later requested discontinuation of further resuscitative efforts. Death was pronounced approximately 40 h after TACE. An autopsy was offered and declined by the family.

**Figure 4:**
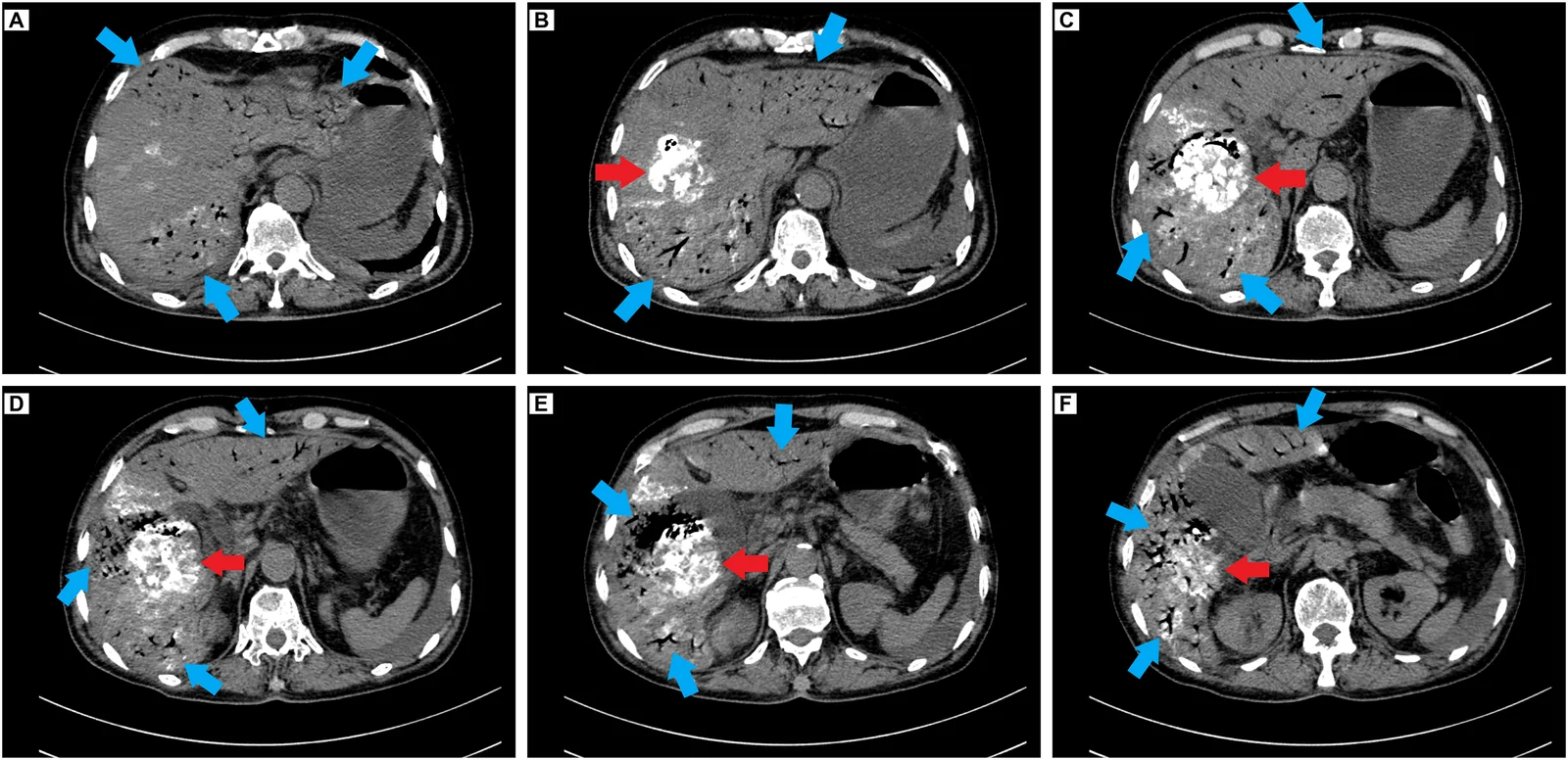
Emergency computed tomography after TACE. **(A–F)** Axial CT images at different levels show peripheral branching hepatic portal venous gas (blue arrows). In panels **(B–F)**, red arrows mark the treated tumors in the right hepatic lobe, which contain indeterminate intratumoral gas-like foci.

## Discussion

This case records a compressed sequence from gastrointestinal symptoms to HPVG, circulatory deterioration, and death after TACE. The sequence may help clinicians recognize a dangerous change in an otherwise common postprocedural symptom pattern. It does not show that TACE or either embolic platform caused HPVG. The temporal association also cannot establish how gas entered the portal circulation or why the patient died.

The course differed from the two previous reports of HPVG after TACE. Xu et al. described HPVG approximately 3 days after a fifth TACE, followed by a culture-positive liver abscess and death (5). Wu et al. reported pre-existing pneumatosis and bowel dilatation before TACE, with HPVG and obstruction on postoperative day 5 (6). That patient recovered with conservative treatment. In the present case, abdominal symptoms began at 21 h, HPVG was identified at 38 h, and death occurred at 40 h. The combined embolization regimen differs from those reports, but comparative evidence of increased risk is absent.

The wider literature also cautions against assigning prognosis from HPVG alone. In a Japanese national inpatient database of 1,590 patients, bowel ischemia occurred in 53% and in-hospital mortality was 27.3% (9). Outcomes varied markedly with the underlying disorder. Other cohorts likewise describe heterogeneous causes, treatments, and outcomes (1–4, 10–12). HPVG should therefore be interpreted with the clinical, laboratory, and CT findings.

A hepatobiliary pathway is possible but unproven. Gas-like foci were present within the treated tumors, but intratumoral gas after TACE does not by itself identify an abscess. Bisseret et al. found intratumoral gas in 5% of tumors examined 4–6 weeks after TACE, and only 3 of 26 gas-containing tumors were associated with abscess formation (13). Those delayed findings cannot be applied directly to CT obtained 38 h after TACE. Iodized oil and other embolic material may also complicate visual classification. The available images cannot distinguish sterile necrotic gas, infected necrosis, abscess, embolic material, or a mixture. Embolization may also cause biliary injury or, after nontarget delivery, gastroduodenal ischemia (14–18). However, angiography showed no reflux or extrahepatic deposition, and no such injury was confirmed.

An intestinal pathway also remains possible. Constipation and bowel dilatation were present before HPVG was identified. Increased intraluminal pressure or ischemic mucosal injury can permit gas to enter mesenteric veins (1, 10, 11). The enema was administered only after constipation and abdominal distension had begun. It could have transiently increased intraluminal pressure and aggravated an already compromised bowel, but the sequence does not support it as the initiating event. No procedural, pathological, or postmortem evidence demonstrated enema-related bowel injury, perforation, or infection. We therefore regard the enema as a possible contributing factor rather than an established cause.

Systemic infection is another possibility, but overwhelming sepsis was not established. Gas-forming infection can introduce gas into mesenteric or portal veins, while sepsis-related hypoperfusion may weaken the intestinal barrier (1, 10, 11). Mortality in the national database reached 56.4% among patients with sepsis (9). Infected tumor necrosis or an abscess could therefore have contributed. However, lactate, a repeat leukocyte count, blood cultures, and other microbiological samples were not obtained after deterioration. No antimicrobial therapy was administered before death, and no postmortem examination was available. These gaps prevent confirmation of infection or sepsis as the initiating or dominant mechanism.

Recognition is difficult because pain, nausea, and vomiting are common after TACE and are often attributed to postembolization syndrome. A meta-analysis of 81 studies and 9,495 patients confirmed that gastrointestinal adverse effects are frequent (19). The trajectory and physiological context are therefore crucial. Persistent distension, escalating pain, repeated vomiting, tachycardia, hypotension, or altered consciousness should trigger immediate reassessment. When feasible, assessment should include hemodynamic examination, laboratory tests for hypoperfusion and infection, and early contrast-enhanced CT. Management should integrate examination, lactate and other laboratory findings, bowel-wall enhancement, pneumatosis, obstruction, dilatation, ascites, and the extent of portal venous gas (1–4, 9–12, 20).

This report has important evidentiary limits. No post-deterioration lactate, repeat leukocyte count, blood culture, or other microbiological result was available. There was no serial imaging, attenuation measurement, endoscopy, surgery, tissue diagnosis, or autopsy. Direct hepatobiliary injury, intestinal barrier failure, bowel ischemia, systemic infection, and nontarget embolization cannot be separated. The cause of the rapid deterioration and death therefore remains uncertain. The practical message is early reassessment of progressive symptoms and physiological decline, not retrospective assignment of causality to the embolization regimen.

## Conclusion

HPVG is a rare but potentially fatal event after TACE. Progressive abdominal symptoms with peripheral branching intrahepatic gas require urgent evaluation for bowel injury, infection, biliary complications, and circulatory compromise. Intratumoral gas-like foci after embolization require cautious interpretation because sterile necrosis, infected necrosis or abscess, and embolic material may overlap. This case cannot establish the mechanism of HPVG or death. It also provides no evidence that combined iodized oil and drug-eluting microspheres are riskier than either platform alone.

## Patient perspective

The patient’s perspective could not be obtained because he died shortly after the rapid clinical deterioration.

## Data Availability

The original contributions presented in the study are included in the article/**Supplementary Material**. Further inquiries can be directed to the corresponding author.
