# Supplementary material for: Case Report: Rapidly fatal hepatic portal venous gas following transarterial chemoembolization for hepatocellular carcinoma
Source: Front Oncol. 2026 Oct 19;16:1951000. doi: 10.3389/fonc.2026.1951000 (PMC13724570; doi:10.3389/fonc.2026.1951000)
Supplement: Supplementary file 1 [file Table1.docx]

# CARE Checklist for Frontiers in Oncology Case Report Submission

Manuscript: Case Report: Rapidly Fatal Hepatic Portal Venous Gas Following Transarterial Chemoembolization for Hepatocellular Carcinoma

Prepared as the completed CARE checklist for supplementary publication; manuscript locations are identified by section headings and figure numbers.

| **CARE item** | **Topic** | **Location in manuscript** | **Notes** |
| --- | --- | --- | --- |
| 1 | Title | title page | The title begins with 'Case Report:' and states the primary clinical focus. |
| 2 | Keywords | Keywords | Six keywords include the article type, intervention, diagnosis, and imaging finding. |
| 3 | Abstract | Abstract | The abstract reports the intervention, clinical course, outcome, unavailable infection-related tests, and evidence-limited interpretation. |
| 4 | Introduction | Introduction | The rewritten introduction defines the clinical problem, identifies the two prior post-TACE reports, and states the evidence boundary. |
| 5 | Patient information | Patient information and clinical findings | De-identified medical, smoking, alcohol, allergy, intervention, transfusion, and family histories are reported, including relevant negative findings. |
| 6 | Clinical findings | Patient information and clinical findings; Follow-up and outcomes | Presenting findings, deterioration, vital signs, imaging, and unavailable laboratory or microbiological data are stated without imputation. |
| 7 | Timeline | Timeline; Figure 1 | A two-level timeline presents symptoms, vital signs, imaging, treatment, outcome, and unavailable evidence at 0, 21, 37, 38, 39, and 40 h. |
| 8 | Diagnostic assessment | Diagnostic assessment; Figures 2 and 4 | Biopsy, immunohistochemistry, MRI assessment of HCC, and CT diagnosis of HPVG are described. |
| 9 | Therapeutic intervention | Therapeutic intervention; Follow-up and outcomes; Figures 1 and 3 | The TACE agents, doses, sequence, particle size, postoperative supportive care, resuscitation, non-use of antimicrobials, and declined ICU transfer are reported. |
| 10 | Follow-up and outcomes | Follow-up and outcomes | The rapid deterioration, treatment, unavailable tests, ICU decision, resuscitation, death, and declined autopsy are described. |
| 11 | Discussion | Discussion | The revised discussion compares prior cases, considers competing mechanisms and the enema, and states the laboratory, microbiological, pathological, and postmortem limitations. |
| 12 | Patient perspective | Patient perspective | The patient's perspective was unavailable because he died after rapid clinical deterioration. |
| 13 | Informed consent | Ethics statement | Ethics approval (202607-H-034), waiver of participation consent, and written next-of-kin consent for publication are documented. |
